# Patient advocacy group perspectives on treatment priorities and clinical trials for the rare neurodevelopmental condition, Prader-Willi syndrome

**DOI:** 10.1186/s13023-026-04253-1

**Published:** 2026-02-14

**Authors:** Anthony Holland, Theresa Strong, Lauren Schwartz, Lynn Garrick, Charlotte Hoybye, Maithe Tauber, Marguerite Hughes

**Affiliations:** 1 Department of Psychiatry, University of Cambridge, Cambridge, UK; 2 Immediate Past President IPWSO, Cambridge, UK; 3 Research Programs, Foundation for Prader-Willi Research, Covena, CA USA; 4 Mental Health Programs, Foundation for Prader-Willi Research, Covena, CA USA; 5 Medical and Research Co-Ordinator, Minnesota, USA; 6 Program Director AME Community Services, and Chair of the IPWSO Professional Providers and Caregivers Board, Minnesota, USA; 7 Research Affiliate, Department of Molecular Medicine and Surgery, Karolinska Institute, Chair IPWSO Clinical and Scientific Advisory Board, Stockholm, Sweden; 8 Professor of Pediatrics, University of Toulouse, Director of the Reference Centre for PWS France, Member of the IPWSO Clinical and Scientific Advisory Board, Toulouse, France; 9 Immediate Past Chief Executive Office IPWSO, Galway, Ireland

**Keywords:** Prader-Willi syndrome, Neurodevelopmental disorder, Neuropsychiatric phenotype, Atypical development, PWS genotype, Clinical trials, Hyperphagia, Temper outbursts

## Abstract

**Background:**

Using Prader-Willi syndrome (PWS) as an example, this position statement focuses on supporting the development and evaluation of new treatments for rare neurodevelopmental disorders. As members of two patient advocacy organisations we have drawn on published research and the experience of participants, parents, paid care providers, and clinicians to make recommendations to improve the translational process from drug discovery to treatment approval and availability.

**Main text:**

PWS is a rare complex genetically-determined neurodevelopmental condition. Severe hypotonia and failure to thrive are apparent at birth, followed by an atypical pattern of development associated with variable but characteristic physical and neuropsychiatric phenotypes. Like other neurodevelopmental conditions, atypical brain development results in intellectual and cognitive impairments. Relative growth and sex hormone deficiencies, the onset of hyperphagia in early childhood, and other phenotypic characteristics are a consequence of impaired hypothalamic function. Although growth hormone treatment for children with PWS has improved the phenotype of PWS, treatments for other features of the disorder are needed given their severe impact on the lives of people with PWS and their families. We identified factors that have delayed the development and evaluation of new treatments including: a limited understanding of causal mechanisms; additional measures required due to the participants’ intellectual and cognitive impairments potentially impacting on consent, compliance, and requiring informant rather than participant-based outcome measures; high risk for co-morbidities; and recruitment from a small population; and over-restrictive inclusion/exclusion criteria.

**Conclusions:**

Clinical trials can be performed in PWS but the associated complexities requires an understanding of the challenges and a more flexible approach to recruitment and trial design. The identification of biomarkers to screen new agents and for prediction of efficacy and outcome are warranted. Novel statistical methods for small numbers and/or qualitative methodologies, may be necessary and appropriate. Robust and meaningful engagement of those living with PWS and their families, advocacy groups and expert clinicians is critical to the successful clinical trial completion. Given the example of the variable availability and affordability internationally of growth hormone to people with PWS, we express concern about the availability of new treatments globally.

## Introduction

This position statement has been prepared on behalf of the International Prader-Willi Syndrome Organisation (IPWSO), and the Foundation for Prader-Willi Research (FPWR) by an expert group of clinicians, researchers, parents, and social care providers. IPWSO is a global charity advocating for the needs of people with PWS across the world. FPWR is primarily based in the USA and advances PWS research both within and outside of the USA. Both organisations also advocate for and support research and clinical trials involving people with PWS.

Identifying new clinical treatments has proved a challenge in PWS, with no new treatments approved for many years. However, this has begun to change with one treatment for hyperphagia recently approved by the Food and Drug Administration (FDA) in the USA and potential new treatments for various aspects of the PWS phenotype now in trial or due to be evaluated. This joint IPWSO/FPWR expert group was established with the aim of preparing a position statement to inform those working in the pharmaceutical industry; clinicians and researchers engaged in, or considering undertaking, clinical trials of medical products designed to treat various aspects of the PWS phenotype; and national and regional regulatory authorities who are responsible for approving any new treatment.

In this position statement we consider the notional pathway to the approval and ultimate availability of new treatments including: the identification of treatment needs, mechanistic studies that inform treatment development, the clinical trials process itself and ensuring access globally to people with PWS according to need. By identifying the challenges in the treatment development pathway, particularly those difficulties associated with clinical trials for a rare disorder such as PWS, we propose solutions, with the aim of improving the process from drug discovery to treatment approval and accessibility.

The opinions stated are the views of the authors informed by published peer-reviewed research, systematic reviews and their experiences of research and clinical trials. All the authors have worked closely with pharmaceutical companies in various roles and also with participants with PWS involved in clinical trials, together with their families and professional carers. The group also liaised with the PWS International Clinical Trials Consortium (PWS-CTC). This consortium was established in 2015 by FPWR and seeks to address unmet scientific, technical, clinical and regulatory needs for PWS clinical trials. The PWS-CTC comprising equal representation from patient advocacy groups, academics and industry, have recently prepared ‘Tips and Tricks’ for clinical trials based on the experience of trials^1^

## Background

PWS is one of an estimated 7000 to 8000 rare disorders, many of which are neurodevelopmental conditions such as PWS. These conditions are usually of genetic origin and brain development is either affected from very early in development or brain function starts deteriorating in childhood [1]. Characteristic of all such rare genetically-determined neurodevelopmental conditions is the manifestation, to varying degrees, of an impaired ability to acquire the same level of functional, communication, intellectual, social and/or educational abilities compared to that of typically developing children of similar ages.

As we discuss in this paper, such neurodevelopmental conditions give rise to particular challenges when undertaking research and clinical trials in a manner that is ethically acceptable to all concerned, including Review Boards, and whose methods will result in findings that are of a quality required of the regulatory authorities. It is essential in any clinical trial that sufficient and appropriate clinical data is obtained to enable regulatory authorities to determine whether or not to a proposed treatment is safe and effective for the intended population, and ultimately to ensure that the treatment is available and clinicians will be willing to recommend and prescribe.

Particularly for families of children with rare disorders, the emergence of a potential new treatment is likely to be a rare event raising expectations and providing hope that quality of life may be better for their affected children. However, it is rarely that straightforward. For example, other rare neurodevelopmental disorders (e.g., Fragile X syndrome [2]), have no approved treatments and others have treatments with limited efficacy and significant side effects (e.g., Rett syndrome [3]). Until recently in PWS the only medical treatment that had been approved was growth hormone replacement for use in children and, in some countries also for adults (see review by [4]). In the last few months Diazoxide Choline Controlled Release (DCCR, marketed as Vykat XR) has been the first and, at present, only treatment for hyperphagia for people with PWS to be approved by the US Food and Drug Administration (FDA). This treatment was evaluated over a period of ten years in a succession of closed and open phase trials, including a blind withdrawal phase [5–8].

With further trials involving people with PWS in progress or planned we reflect, from the perspectives of our two organisations, on the experience so far. Whilst there are undoubted challenges to the development and testing of what are hoped will be new effective and safe treatments for this particular rare neurodevelopmental condition, we consider there is also reason to be optimistic. The development and testing of treatments for rare disorders has increased markedly and in 2024 the Pharma Annual Review reported that just over 31% of drugs being tested were for rare disorders^2^

## The PWS genotypes and variations in the phenotype

PWS is a rare, complex, genetically-determined neurodevelopmental condition with a characteristic physical and neuropsychiatric phenotype that arises as a consequence of the absence of expression of a contiguous set of paternally-expressed/maternally imprinted genes on chromosome 15q11-13 [9] updated 2024). The three main causes of PWS are: a small de novo deletion on the paternally-inherited chromosome 15 (different sized deletions have been reported) at the 15q11-13 locus (delPWS); uniparental maternal disomy (mUPD) where both chromosome 15s in the affected individual are of maternal origin and the paternal copy is lost at conception; and an Imprinting Centre Defect (ID). The birth incidence of PWS has been estimated in various studies as being in the order of 1:20,000 [10, 11]. Reported population prevalence has been variously estimated to be between 1:37,000 and 1:54,000 with similar rates according to sex [11, 12].

In utero reduced foetal movement is reported by mothers. Post natally, extreme hypotonia, low weight for gestational age and immature development of the external genitalia (undescended testes in boys and small labia in girls) are common. This early phenotype is also characterized by poor sucking and swallowing functions, and an apparent poor appetite often leading to failure to thrive requiring the use of a feeding tube. In the paediatric population, most of the deaths occur before the age of two years and are mainly due to respiratory causes [13]. The subsequent onset of hyperphagia in childhood can result in extreme levels of obesity if food is not restricted to a level commensurate with their energy and growth needs.

In childhood, in addition to hyperphagia, there is also evidence of developmental delay and delay in the acquisition of intellectual, cognitive, social, educational and functional abilities [14] for review). Levels of IQ have been found to be shifted downwards by an average of 40 points together with impaired social cognition, and a reduction in cognitive flexibility. There are increased risks for the development of various behaviours of concern, including temper outbursts, severe skin picking, and repetitive and ritualistic behaviours, together with emergence of affective instability and situational anxiety [15]. Reduced growth (unless growth hormone treatment is being given) and impaired sexual development due to relative growth and sex hormone deficiencies of hypothalamic origin, respectively, are evident and these, together with other phenotypic characteristics, are likely a consequence of impaired hypothalamic development [16, 17].

## The need for new treatments

Advances in our understanding of the needs of people with PWS have been considerable. Where early diagnosis and guidance to families is available and ongoing support is provided, the picture of the phenotype may be very different. For example, severe obesity is less apparent if a child grows up in a food managed environment, where they and their families have an understanding of the need to manage access to food and the capacity to take the practical actions needed to do so. Now that an early diagnosis can be expected in countries with advanced healthcare systems, information for parents, early multidisciplinary support, and the use of growth hormone supplementation have become important interventions. With improvements in life-expectancy there is an increased focus on managing transition to, and needs in, adult life (see [10, 18–20]).

However, the challenge is that the maintenance of such positive influences can be fragile. If, for example, the support environment changes because of parental or other caregiver illness subsequent life trajectories may deteriorate dramatically. Therefore of particular concern to families and for people with PWS is the development of new treatments particularly for those behaviours and feelings, such as hyperphagia, anxiousness and temper outbursts [21, 22] that can make independence for people with PWS very problematic, particularly in adult life. For example, whilst hyperphagia has a clear biological basis to it, whether it results in life-threatening level of obesity is at present dependent on the quality of the support environment. Where food security cannot be maintained, severe obesity is highly likely. This is associated with increased morbidity and mortality, including type-2 diabetes mellitus, cardiovascular and respiratory co-morbidities, and sleep apnoea (see systematic reviews by [23] and [24]).

Surveys of families have documented the profound impact of having a child with PWS on the families health and wellbeing [25, 26], and also the tremendous unmet clinical need [21]. The neuropsychiatric and cognitive profiles associated with PWS give rise to a level of burden for the primary caregiver that is higher than that experienced by those caring for individuals with Alzheimer’s disease or stroke [27]). This burden rises with increased hyperphagic behaviour [28]. Any cost-benefit analysis with respect to clinical trials needs to acknowledge that the absence of treatments, particularly for hyperphagia and behavioural problems, comes with high emotional, financial, and societal cost for all concerned. The reasons include increased number of hospitalizations, high utilization of adjunctive therapies, and increased length of stay in hospital made worse by the lack of adequate follow up care to maintain any gains they may have made during hospitalization [29].

## Disease modifying and symptomatic targets for therapy development in PWS

Our aim in this section is to give a brief overview of the working hypotheses that inform our understanding of particular aspects of the PWS phenotype listed above, some of which are targets for treatment development. For specific single gene neurodevelopmental conditions, mechanistic studies focus on identifying the function of the relevant gene and the missing or atypical gene product [30] (see for discussion of the translation cycle for genetically-determined neurodevelopmental conditions). However, in PWS this approach has been limited by the fact that there remains some uncertainty as to the precise gene(s) that are central to understanding the condition and how the absence of expression of these maternally-imprinted coding and non-coding genes located at 15q11-13 relates to the phenotype (see [31] and [32] for role of SNORD116). Investigation of genotype/phenotype relationships and identifying target pathways has largely been dependent on rare cases of people with PWS with unusual genetics [33], the use of different genetic mouse models of PWS (see [34] for review, also see [35] with reference to oxytocin research) and more recently on induced pluripotential stem cells and brain organoids [36].

Given the presence of the early phenotype at birth and the main early and late phenotypic characteristics associated with PWS, one focus of clinical research has been on studying specific neural systems that are likely to be relevant. These have included: the hypothalamus and its projections, oxytocin networks in the brain, and peripheral and central appetite and satiety pathways and central reward networks, and more recently the autonomic nervous system. An additional complexity impacting treatment development is that to be successful interventions may have to take place at critical times in development when there is increased neural plasticity and the brain is at its most responsive [37].

Potential treatments for various aspects of the PWS phenotype are considered below and are summarised in Table 1. Some of these treatments aim to be disease modifying in that, if effective, they would alter the subsequent course of development and have a permanent effect. Other treatments are symptomatic targeting specific aspects of the PWS phenotype. Some of these symptomatic treatments may also have disease modifying effects. For example, growth hormone treatment given to improve short stature also, particularly when starting early in childhood, has a modest beneficial effect on intellectual functioning and on maintaining such cognitive abilities in adult life [54, 55].

**Table 1:** Unmet needs for individuals with PWS, examples of potentially disease modifying and symptomatic treatments for PWS, and potential methods of assessment

Unmet need	Potential Intervention	Symptomatic or Disease modifying	Aspect of phenotype to be assessed	Potential Clinical Outcome Assessment	Comments/references
**Developmental delay/cognitive deficits**	Treatment to re-activate imprinted maternal copies of paternally imprinted genes at 15q11-13	Disease modifying	Cognitive development IQ Adaptive function	Mullen scales of early learning NIH Toolbox Cognition Battery – adapted for ID (NIHTB-CB) WISC-R/WAIS-R Vineland Adaptive Behavior Scale-3 (VABS-3)	Interventions early in life may impact developmental trajectory [38] [39] [40–42]
**Failure to thrive**	Oxytocin or its analogue given early in development. Early intervention protocols	Disease modifying	Transition to feeding competency	Nutritional Phase evaluation Swallow study	
**Social skills**	(Oxytocin), Behavioural programs: PRETEND, BOSS	Disease modifying	Social competence	Social communication Questionnaire (SCQ) Social Responsiveness Scale (SRS)	[43] [44]
**Hyperphagia**	Medications or neuromodulation approaches acting of peripheral and/or central feeding pathways	Symptomatic Possibly disease modifying	Reduction in hyperphagic behaviours	Hyperphagia Questionnaire-Clinical Trials (HQ-CT) other scales measuring problematic food behaviours Food Safe Zone (FSZQ)	Treatment of hyperphagia early in life may have longer term benefits resetting feeding pathways
**Temper outbursts**	Medications or medical device with mode of action on the ANS	Symptomatic	Frequency and/or intensity of temper outbursts	Aberrant Behaviour Checklist −2 (ABC-2) PWS Profile Modified Overt Aggression Scale	[45] [46]
**Anxiousness**	Behavioural medications; behavioural interventions such as ABA	Symptomatic	Behaviours associated with anxiousness	PWS Anxiousness and Distress Questionnaire-PADQ	[47]
**Other behaviours:** OCD/repetitive behaviours/rigidity	(centrally acting behavioural medications; ABA or other behavioural intervention)	Symptomatic	Characteristic behaviours associated with PWS	RBS-R PWS Profile	[48] [49]
**Self-injurious behaviour**		Symptomatic	Skin picking lesions	Self-Injury Trauma Scale	[50]
**Obesity**	Anti-obesity drugs	Symptomatic	Weight Metabolic markers	Weight loss, BMI, Metabolic markers, gut hormones	
**Disrupted sleep/excessive daytime sleepiness**	Wake promoting medications or devices; interventions regulating circadian rhythm	Symptomatic or potentially disease modifying	Sleep quality and daytime sleepiness	Epworth Sleepiness Scale Wearable sleep trackers	[51] [52]
**General well-being**	For any PWS treatment	Symptomatic	Quality of life, Impact of PWS on daily life	PROMIS Life Satisfaction-short form 8b & others Clinician rated severity/improvement Caregiver rated improvement	Caregiver burden can be a proxy for improvement [53]

### Disease modifying interventions

- Genetic modification: reactivating the maternally imprinted genes whose absence of expression are associated with PWS has the potential for the prevention of the phenotype that is characteristic of PWS. These are under consideration using PWS mouse models [56]. However, many challenges remain to establish that a genetic therapy would result in improvements in the phenotype of individuals with PWS, but the field has been evolving rapidly and clinical experience with other neurodevelopmental disorders may help guide development in PWS.
- Neonatal hypotonia, severe sucking and swallowing impairments in infancy: the extreme hypotonia present at birth, inability to feed and the associated failure to thrive requires expert intervention and in some cases intensive paediatric care. There is the possibility that the condition of the baby in-utero [57] and the lack of treatment for the sub-optimal post-natal physical state of the infant may have longer term effects on the child’s developmental trajectory and their health (see [35, 58]). Ongoing clinical development programs are evaluating the concept of a central neural deficiency and, for example, the role of the neurotransmitter oxytocin. One possibility is that intranasal oxytocin for infants with PWS may rescue the neonatal phenotype (sucking/swallowing and social impairments) as well as having a longer term beneficial effect, such as improved motor development, providing it is given during a critical period in early infant development.
- Early phenotype and social development: the interest in oxytocin in PWS has its origins in an earlier observation of a 46% reduction of oxytocin-secreting neurons in five post-mortem samples of hypothalamic tissue from people with PWS [35, 59]. An alteration in the oxytocin-secreting neurons was also described in two relevant animal models of PWS, the Necdin KO mouse and the Magel2 KO mouse. In adult mice the administration of oxytocin postnatally restored sucking and normalized learning, memory and social cognition by rescuing the development of the oxytocin system [60, 61]. Studies of oxytocin and its receptors in the brains of the Magel 2 knockout mouse model of the PWS-related condition, Schaaf-Yang Syndrome [62], found that early post-natal administration of oxytocin prevented some cognitive and autistic-like features developing. These animal and preclinical findings paved the way for the clinical use of early oxytocin treatment in neonates and infants with PWS with the aim of rescuing the oxytocin system and impacting the trajectory of the neurodevelopmental disorder [35, 58, 63].

### Symptomatic treatment targets

#### Hyperphagia

At present the mechanisms that underpin the risk of hyperphagia in people with PWS remain uncertain. As the role of peripheral and hypothalamic feeding pathways have become better understood a distinction has been made between appetite and hunger. The former is seen as underpinned by reward pathways in the brain and relating to hedonic aspects of food choice and eating behaviour and the latter with satiety pathways and survival orientated homeostatic processes [64]. Largely based on neuroimaging findings with respect to PWS some have argued that dysfunction of appetite and reward value of food is central to understanding hyperphagia [65] and others that a failure of satiety is the primary deficit in PWS [66, 67]. Brown et al. [16] reported that people with PWS had a developmentally small hypothalamus compared to aged-matched and obesity matched control groups, possibly accounting for the ‘hypothalamic phenotype’ of PWS, including hyperphagia.

Observations made some years ago of raised levels of the orexigenic hormone, acylated ghrelin, in the blood of people with PWS [68, 69] led to this being proposed as a mechanism to explain hyperphagia. This observation led to trials of an unacetylated ghrelin analogue (Livoletide) given to reduce active ghrelin in the brain. However no significant benefit was found in one study [70], although positive findings had been reported in an earlier study [71]. In an open label exploratory phase 2 trial, the MC-4 receptor agonist Setmelanotide showed reductions in BMI and in HQ-CT scores. In a Phase 3 trial of the MetAP2 inhibitor, Beloranib, a reduction in fat mass and weight and a downward shift in hyperphagia scores were observed [72]. In this case it was the observed pulmonary embolism risk that resulted in the trial ending when it did (see [63] for review of trials).

Roof et al. [73] reported on the findings from a Phase 3 trial of intranasal carbetocin given three times daily, with a placebo, 9.6 mg and 3.2 mg arms. In the 3.2 mg arm, significant improvements were observed in hyperphagia and in general functioning, but significant efficacy was not demonstrated for the 9.6 mg dose. Recently the findings from a Phase 3 trial of Carbetocin for the treatment of hyperphagia were reported. Intranasal carbetocin did not demonstrate a statistically significant improvement over placebo on the study’s primary endpoint, change from baseline to Week 12 on the Hyperphagia Questionnaire for Clinical Trials (HQ-CT), nor was there separation from placebo on any secondary endpoint.

Diazoxide Choline Controlled Release (DCCR), the one medication that has been approved by the US FDA for treating hyperphagia in PWS, acts on K_ATP_ channels in the orexigenic NPY/AGRP pathways in the hypothalamus, reducing secretion of appetite stimulating hormones. This medication has also been reported to result in improvements in behaviour [7, 74]. Importantly, other trials of different agents are also underway or are planned that act on alternative pathways. These include agents acting on bitter-taste receptors in the gut wall impacting on local cholecystokinin (CCK) release, cannabinoid 1 receptor, and adrenergic and serotonergic cerebral pathways.

At present the recommended approach to treatment remains the prevention of the consequences of uncontrolled hyperphagia – severe obesity and the secondary obesity-related co-morbidities – through ensuring that access to food is strictly controlled often by physical means, such as locked doors and cabinets, and/or through supervision. However, the need for restrictions on access to food and, for example, the subsequent restrictions on the freedom to travel independently impacts life opportunities and quality of life. The present impact on the lives of people with PWS, the level of risk to physical health, and the impact on quality of life that is associated with there being no treatments for hyperphagia are therefore very significant. Parents, not surprisingly, express a high tolerance of risk for treatments that would reduce hyperphagia in PWS, understanding the high risk of morbidity and mortality in the untreated state [75].

### Other behaviours of concern associated with PWS

For other aspects of the PWS phenotype, such as temper outbursts, skin picking, impaired cognition, repetitiveness, and anxiousness, there is still not a full understanding of causation.

- Temper (emotional) outbursts: there are no approved treatments for such outbursts, which affect in the order of 60% to 80% of people with PWS during their lifetime [15]. These outbursts may be limited to shouting and crying, or extend to the breaking of objects and attacks on others, with outbursts lasting from minutes to hours [76]. There have been open label trials of antipsychotic [77] and antidepressant medications [78] reporting some improvement in behaviour. A recent double blind placebo controlled trial of guanfacine slow release, an alpha-2A adrenergic receptor agonist, reported significant reductions in aggressive behaviour, as measured using the Aberrant Behavior Checklist and the Modified Overt Aggression Scale, and also a significant reduction in the number of skin-picking lesions [79]. Guanfacine may have its affect by reducing sympathetic nervous system activity. Significant reductions in temper outbursts were also reported after some months of vagus nerve stimulation from an implanted stimulator [80], a finding subsequently replicated in a small trial using transcutaneous vagus nerve stimulation (tVNS) [81]. In the second of these two studies normalization of heart rate variables were found to correlate with improvements in behaviour [82]. tVNS is now being investigated in a larger trial undertaken by FPWR. From a mechanistic perspective there is a coming together of evidence to support the hypothesis that autonomic nervous system dysfunction is a key factor moderating the risk for temper outbursts [83, 84].
- Anxiousness and repetitive behaviours: Individuals with PWS frequently show high levels of anxiousness, which presents differently from anxiety in the general population, but which can have a significant impact on quality of life [21, 22]. Repetitive questioning and other repetitive behaviors may also negatively impact social interaction and development. Pharmacological interventions (e.g., oxytocin) and behavioral interventions have thus far had limited impact, however, mitigating these aspects of the PWS behavioral phenotype remains a high priority for the PWS community.
- Self-injurious behaviour: this is usually in the form of severe skin or rectal picking. Whilst this behaviour does not put others at physical risk it severely impacts on the person with PWS and may cause significant emotional distress to those around them. The presence or not of such behaviour has been shown to be influenced by environmental factors [85] and at its most severe it results in breakdown of the skin, severe bleeding, and a high risk of infection. One small open label trial of topiramate [86] and another of N-acetyl cysteine both reported some benefit [87]. A second small open label trial of topiramate suggested some clinical benefits both with respect to self-injury and also mood stabilisation and hyperphagia [88]. However, there have been no gold standard trials.
- Sleep disorders: sleep disorders are very common in people with PWS, including central and obstructive sleep apnoea, other night-time sleep disorders, and cataplexy. Excessive daytime sleepiness may affect as many as 90% of people with PWS and its presence is associated with the occurrence of depression, anxiety and psychosis [89]. Whilst obesity and the subsequent development of obstructive sleep apnoea is a major concern, central sleep apnoea is also observed. A proof of concept trial of pitolisant showed benefit in those receiving the highest dose [90], and pitolisant is at present being evaluated in a Phase 3 trial for the treatment of excessive day time sleepiness in the absence of sleep apnoea.
- Other areas requiring research and intervention in people with PWS include: the evaluation of early educational programs and their impact on subsequent social functioning and behaviour [91]; strategies and treatments to mitigate the impact of developmental delay/Intellectual disabilities, cognitive rigidity, and impairments in social cognition; management and treatment of scoliosis; management of severe and potentially life-threatening gastroenterological conditions, such as severe constipation, delayed gastric emptying, gastroparesis, and gastric rupture; psychiatric medications for treating affective psychosis, which predominately affects those with mUPD; and assessments and treatments for possible disorders of later life, such as dementia.

In the wider context of mechanistic studies and treatment development it is also important to recognise that various forms of psychological intervention (for example, applied behavioural analysis), are also treatments and, furthermore, the quality and nature of support available to a person with PWS and their family may also be considered to be part of a ‘treatment package’. Informed support and management of the food environment reduce the frequency and severity of outbursts [92].

## Undertaking clinical trials involving participants with PWS

The involvement in research and clinical trials of people with intellectual impairments, those with other cognitive and/or social impairments, and/or who live under circumstances where others may have near total control over their lives, has in the past included exploitation and abuse. As a result the World Medical Association’s Declaration of Helsinki, first published in 1964, established the ethical principles for medical research involving human subjects. These included respect for self-determination and the right to be informed as well as particular safeguards for those seen as potentially vulnerable under such circumstances (see [93] for discussion of vulnerability in a research context). From a contrasting perspective, that of equality, people with a rare disorder, such as PWS, also have a right to expect that methodologically sound and ethical research will be undertaken so that new effective and safe interventions can be developed that will improve their lives.

The gold standard for such trials are for them to be placebo-controlled with matched active treatment and placebo groups, with agreed primary outcome measures, and sufficient statistical power to ensure that where there is a treatment effect that can be detected However, in the case of rare conditions, a major challenge, in the first place, is to engage the necessary range of expertise and establish the funding to develop potential treatments and/or to identify medications that may be repurposed and go to trial. There are then unique challenges to undertaking the trials and finally there is the challenge of ensuring access globally to any new treatment if it is approved were there are limited financial resources. The above issues and potential solutions, are summarized in Table 2, with specific aspects considered in greater detail below.

**Table 2:** Enhancing clinical trials and treatment development for a rare condition

**Challenges** Dispersed, rare population Incomplete understanding of natural history of the disorder, unmet medical needs and treatment targets Complex behavioural phenotype, impacted by environment Presence of comorbidities with potentially complex medication use Impaired cognition of potential participants, impacting capacity to consent and ability to report on treatment effects Lack of validated clinical outcome assessments that capture phenotypes of interest Lack of awareness of the disorder among pharmaceutical industry and regulatory scientists **Solutions** Raise awareness of syndrome Establish and maintain partnerships between researchers, pharma, clinicians, and the PWS patient community Document unmet needs, assess disease impact and identify patient-centred treatment priorities Support research characterising natural history, treatment targets and possible causative mechanisms Develop outcome measures that are fit for purpose, given the cognitive impairments and unique characteristics of children and adults with PWS Leverage advocacy groups to educate the patient community about clinical trials and facilitate trial recruitment Educate pharmaceutical and regulatory scientists about the patient experience and unmet medical needs Engage families, expert clinicians and advocacy groups early in protocol development to tailor protocol for success Consider adjustments to standard trial protocols given the need for informant support, the impact of cognition on capacity to consent, and the challenges of travel for in-person assessments Work in partnership to enable socioeconomic assessments that determine value and evaluate risks and benefits of new treatments in the ‘real world’ Strengthen international ties among advocacy groups, share information and strategies to support approval globally and achieve equitable access to new treatments	

**Key Stakeholders**

Advocacy organisations representing people with PWS and their families Clinicians and academics with understanding of and interest in the needs of people with PWS

Pharmaceutical companies

Regulatory Scientists

## General trial related factors: family and investigator experience

As a project conducted by the PWS-CTC, families of individuals with PWS who had taken part in at least one trial were interviewed, as were principal investigators and study site coordinators at several sites in the USA and one in Canada. The aim of the interviews was to obtain feedback on the experience of participating in PWS clinical trials. The findings are given in two reports ‘Improving the Clinical Trials Experience: Recommendations from Principal Investigators and Study Coordinators’ and ‘Improving the Clinical Trials Experience: Results from a Caregiver Survey of PWS Clinical Trials Participant Experiences’. For principal investigators and study site co-ordinators several factors led to success in conducting and completing the trial including: study site clinicians ‘believing’ in the drug, a feasible and realistic protocol, having access to funding for trial start-up costs, being seen as a collaborator in the trial and being part of a team with other trial sites. Other important factors included: support from advocacy organisations and sufficient costs to cover time and travel costs of families, virtual visits where possible, the use of qualitative data to augment the Hyperphagia Questionnaire for Clinical Trials (HQ-CT) data, and opportunities for junior faculty to be involved.

The other Report focused on family participants, and 74 caregivers, who had participated in at least one clinical trial, completed an extensive survey regarding their experiences in a PWS trial(s). They highlighted the following as key factors for deciding to have their loved one with PWS participate in a clinical trial: knowing that participation may lead to a new treatment, feeling comfortable with the study site team, having questions answered and an acceptable trial schedule, knowing participation would mean having access to try a new drug, having the person with PWS interested in taking part, having a site close to home, and the availability of funding to cover costs (e.g. travel). The greater the distance needed to travel, the number of invasive tests, time away from parental work due to the number and length of visits, were factors that might deter families from participating in a trial. Parents also highlighted that feelings of making a contribution to a better future for people with PWS, being part of a team and having a voice in the clinical trial process were also important.

## Specific factors relating to having the rare condition, PWS

The following are factors, sub-divided under specific headings, which lead to additional complexity and increased costs, and may result in a failure to complete the trial, or impact the way the data can be interpreted. Potential ways of ameliorating the problems are considered in the final section of the paper.

### The rarity of PWS

- Recruitment: There is a cohesive and supportive global PWS community comprising PWS associations in 47 countries, as well as IPWSO and FPWR. This infrastructure has helped families become aware of trial opportunities and significantly aided recruitment. However, the rarity of PWS and the need in some trials to sub-divide between genetic types, sex, or age groups, or control for medication use etc. still makes recruitment difficult. With any rare disorder there is therefore a tension between findings from power calculations for such trials and the feasibility of the resultant requirement to recruit the numbers such calculations indicate. In the case of trials studying treatments that need to be given shortly after birth there is the particular difficulty of being limited to newborns with PWS – for example assuming an average of 700,000 births in the UK per year and a birth incidence of 1:20,000, only 35 births would be expected each year. Also there are likely to be regional differences in the time taken to diagnosis, and there is the ethical issue relating to seeking consent from the parents at a time when they are caring for a struggling baby and are reflecting on what the diagnosis means for them. Recruitment strategies will depend on local circumstances, the existence or not of specialist clinics or specialist centres of reference, and the willingness of clinicians to advocate for the trial. Support from National PWS Associations is crucial in terms of publicity and encouraging families to consider taking part.
- Geography, global spread, regulatory factors: to obtain the necessary numbers for a trial, recruitment will very likely be over a large geographic area and be multisite, and in many cases multinational, adding complexity and cost. For families, time taken to travel and undertake the assessments, having time off work, and coping with the challenges of travel with a child with PWS may all impact on their decision to take part. With international trials there may be the need to translate and validate assessments in other languages and cultures. Other factors, such as conditions related to requirements of one or more of the relevant regulatory agencies may also reduce the pool from which recruitment is possible. For example, in a recent trial there has been the additional requirement that the HQCT informant questionnaire used as the primary outcome measure must only be completed by an informant who is with the participant seven days a week. This rules out the inclusion of participants with PWS who live in group homes or other professional care settings outside of the family. The regulatory authorities required this to be the case as it was under such conditions that the questionnaire had originally been validated.

### The specific characteristics of participants with PWS

- Consent, capacity to consent and compliance: Intellectual and cognitive impairments and the impact of feelings of hunger and a strong desire to seek food are all known to be associated with having PWS. These may impact a person’s capacity to understand the nature, risks and benefits of a trial, to balance the options, and to make a valid decision about whether or not to take part, and to communicate a choice. For children, it is for parents to consent and their children with PWS to assent. For adults (aged 16 or 18 years and over), it would normally be for them to decide. The extent to which the decision is left to the person with PWS, or to a parent or an official Guardian will depend on the policies and laws of the country, or for example, in the European Union, the guidance in the EU Clinical Trials Directive. Whether involving children or adults with PWS in a trial, it is both good practice and is also likely to improve compliance if efforts are made to ensure that the participant with PWS understands what will be asked of them. This may include the use of easy to read material, other media such as video, repeat opportunities to ask questions, and ongoing explanations of what is involved at various times during the course of the trial. Family members or paid support workers are crucial in such situations, both in terms of helping the participant with PWS to understand the trial processes and procedures and supporting the participant throughout the trial to maximise their retention and compliance to the conditions of the trial.
- Co-morbidities: as described earlier, people with PWS are at high risk for particular physical co-morbidities, such as diabetes mellitus, sleep apnoea, severe constipation; and for behaviour problems and mental ill-health, such as emotional outbursts, anxiousness, affective instability and major mental illness. Some will be on medication or start on medication for one or more of the above during the trial. Some interventions for a co-morbidity may counter the treatment being evaluated, or may impact on the effectiveness of the medication being tested (e.g., antipsychotic medication increases appetite, potentially impacting on the efficacy of treatments of hyperphagia). Therefore, the onset of, or deterioration in such co-morbidities and their treatments may in themselves directly affect the outcome of the trial or the interpretation of the data. In addition, the onset of severe physical or psychiatric comorbidities may result in the person not being able or willing to continue, being admitted to hospital, or feeling increasingly stressed and perhaps resulting in family and/or placement breakdown.
- Detection of side effects: people with PWS may be reluctant to report the onset of some new symptom or fail to appreciate the need to report the onset of a new symptom. People with PWS have a high pain threshold, impaired temperature regulation and a reduced vomiting reflex, all of which means that the manifestation of potential side effects of treatment and the onset of developing pathology may be difficult to recognize.

### Support context, and family and social care environment

- Caregiver burden: people with PWS typically need significant caregiver support to learn about a new trial, sign up for a trial, and complete the requirements of a trial. The vast majority of people with PWS of all ages live in their family home and are reliant on family caregivers to support their participation in a trial. Caregiver burden is high in families of people with PWS [27] and the added workload associated with trial participation may be unsustainable for caregivers. In addition, because of their son’s or daughter’s behaviour, families may be anxious about travelling. Repeat clinic visits for blood tests, the extent of the questionnaires repeated over time, and extensive and laborious data collection are factors that may affect recruitment and ongoing participation in a trial. As much as parents might want to support a trial, the above can make them reluctant. Even in the case of the minority of people with PWS who live in residential settings who may want to participate in a trial, staffing levels are often not sufficient to support their participation. All these factors potentially impact on the ability to recruit from residential settings.
- Impact of the environment: the environment in private homes and residential settings and the quality of support available to people with PWS may have a significant impact on the occurrence of particular behaviours, such as temper outbursts, and/or on the consequences of such behaviours (e.g. severe obesity if access to food is not controlled). Whilst, for example, changes in weight or body composition or increase in the extent of scoliosis may be directly measurable, levels of behaviour and mental ill-health may be inconsistent over time, difficult to reliably measure, and affected in either direction with environmental changes, such as the presence of new support staff. Whilst such background variations may be equally distributed across the different arms of any trial, there is the potential for such factors to influence trial outcomes. For example, in the trial of DCCR undertaken by Soleno Therapeutics the onset of restrictions relating to the Covid pandemic were reported to impact outcomes [7].

### Factors specific to the trial design

- Clinical outcome assessments (COAs) and trial endpoints: using a best worst scaling instrument, Tsai at al [22] investigated the priorities for treatment development considered by 457 caregivers who were supporting individuals with PWS ranging from 4 years to 54 years of age. Reductions in hyperphagia and in anxiety were rated as the most important. The choice of primary and secondary outcome measures are a challenge particularly where trials include assessments of hyperphagia, mental state, behaviour and quality of life. Validated COAs that effectively assess the unique behavioural features of PWS being targeted for treatment may not be available. To that end, several PWS-specific COAs have been developed in recent years to address this concern [47, 49, 94, 95]. Conversely, validated COAs may exist but may have been developed in other populations. These would need to be assessed for suitability in the PWS population (e.g. [96]). Ideally it is for participants in the trial to complete such outcome assessments but this may not be possible because of the degree of the participants’ intellectual, cognitive and language impairments or because they fear there will consequences depending on their responses. For this reason, assessments by informants, who have known the person well, may be required [97, 98]. The concern is whether informants, however well they know the participant, are able to give a reliable and valid assessment of, for example, the participant’s mood. Where different informants are involved there are also likely to be concerns about inter-rater reliability.
- Assessments of hyperphagia: the severity of hyperphagia and the risk of severe obesity and the obesity related complications if access to food is not controlled, means that families may have complete control over their child’s access to food. In adult life individuals with PWS may live in a house where their access to food is also controlled. For this reason weight and BMI may be poor outcome measures, because in such environments obesity is unusual as diet is strictly regulated. The primary outcome measure has to focus on measuring reductions in the behaviour of hyperphagia itself. The primary outcome measure for most PWS clinical trials has therefore been the Hyperphagia Questionnaire for Clinical Trials (HQ-CT) [95], based on a PWS-specific measure of hyperphagic behaviours [99]. This is a nine item, well-validated questionnaire completed by the primary caregiver. However, some of the items may not be applicable for all participants because of the lack of opportunity to engage in such behaviours in food restricted environments. This may, therefore, give a score that does not fully reflect the likely severity of the hyperphagia [100]. Furthermore, the difficulty for some parents/caregivers is that they live in a stressed and “abnormal” environment and each person’s definition and understanding of hyperphagia may be a little different depending on their experience. Nonetheless, the HQ-CT has demonstrated the ability to reliably capture PWS-associated hyperphagic behaviours and shows sensitivity to change in clinical trials of investigational agents that impact hyperphagia, supporting its use for PWS clinical trials. Further, the recently developed “Food Safe Zone” questionnaire can provide insight into the level of food access available in the environment of the person with PWS [49].
- Efficacy and effectiveness: trials establishing the efficacy of a treatment and studies demonstrating that the same treatment works well in the real world (effectiveness) are both important. As described above measuring the efficacy and effectiveness of a new treatment is very likely to depend on the reports of others because people with PWS have difficulties giving accurate accounts of their behaviour, mood, and cognitions, such as hunger. Other options, usually as secondary outcome measures, include the use of daily diaries to record outbursts and mental states, qualitative interviews [101], and more recently, data obtained using wearable devices and the use of app-based technologies [102]. Each have their advantages and disadvantages. Daily dairies of emotional outbursts completed by care workers used in one study [81] had the advantage of being closer to being a measure of effectiveness, rather than just efficacy, but staff were not always reliable at completing the necessary daily record. There have been no studies yet that demonstrated the utility of measurable blood-based, physiological, or neuroimaging biomarkers of hyperphagia that might be used as supportive outcome measures. Work in this area is on-going.
- Cost of trials for rare disorders: in an analysis of data collected as part of the KMR Group Clinical Trials Cost Study undertaken in 2016, Martin et al. [103] identified the average costs of trials and also those factors that most impact on costs. As expected, participant numbers and number of sites and visits were significant factors in increased costs. In addition, the greater the number of countries involved and the use of emerging markets also increased costs, with the latter also increasing trial duration. The authors calculated that each additional month for Phase 3 trials costs an additional $671,000. Trials involving rare diseases were not specifically associated with higher cost but the cost per participant was greater due to smaller numbers and more complex trial designs. A further factor contributing to trial costs were inefficiencies within operations across the industry, with companies conducting trials in rare diseases tending to be less efficient. With the expectation from regulators that evidence be based on a gold standard trial design, the pressure to recruit sufficient numbers will mean more sites globally, and mounting costs. One company undertaking a trial in PWS ceased their trial early, before there was clear evidence one way or the other of efficacy, because of the anticipated financial costs of a full trial.

## Conclusions and recommendations

This paper is primarily concerned with trials involving the use of pharmaceutical treatments to treat particular aspects of the PWS phenotype. Such treatments require formal approval by the relevant authorities, such as the European Medicines Agency. With illnesses, such as cancer, the consequences of not treating are usually severe and the benefits of treatment are potentially life-saving, life-extending or positive in reducing pain and level of disability. We propose that in the case of PWS, the same issues and urgencies apply. In countries where there may not be the paediatric and nursing expertise or the necessary diagnostic facilities, infants with PWS may not survive the initial failure to thrive phase. From early childhood hyperphagia and other aspects of the phenotype impact significantly the life expectancy, level of independence, and quality of life of the individual with PWS, and also impacts their family and support costs. Whether it is the early hypotonia and failure to thrive or the hyperphagia, having no treatment available has serious consequences.

Professionals, family members and people with PWS all describe occasions when participants with PWS have been fully engaged and committed to a particular research project or a clinical trial. There are strong and generally very positive relationships between all parties involved in trials in PWS that have taken place or are being undertaken. It is clear that well conducted trials are possible but we argue that there are challenges, and being aware of these, such as those described in this article, may mean that they can be avoided or more effectively managed. When effective treatments for specific aspects of the PWS phenotype are approved they are likely to be transformative in the same way that they can be for cancer and therefore trials are essential. We make the following recommendations:

### The need for more clinical and translational research

Whilst the aspects of the phenotype in need of treatments in PWS are clearly identified, limited understanding of underlying brain mechanisms makes the choice of the appropriate targets for treatment problematic. There is a need for more fundamental translational research, which might in turn result in a more informed choice of treatments to be studied. A detailed understanding of the genetics and epigenetics, the use of genetically-modified animal models, tissue models derived from stem cells, and advanced neuroimaging (see example in autism [104]), are all examples of relevant techniques (see [30]). In this respect research meetings and workshops are critically important so that diverse perspectives can be brought together. Both FPWR, IPWSO and National PWS Associations have central roles in organising such meetings and sustaining the diversity of research into PWS. Funding partnerships between those undertaking the trial and clinical and academic researchers should be encouraged. These convenings can also serve as an opportunity for co-learning between families of those with PWS, scientists, clinicians and pharmaceutical industry scientists, to ensure that the unmet needs and treatment preferences of the PWS community are considered fully in the development of research priorities.

An area of research opportunity for the PWS field is the development and validation of behavioural, blood-based, and neural markers predictive of efficacy. Particularly with respect to hyperphagia, a combination of behavioural observations in structured food environments [105], functional MRI neuroimaging of satiety and reward neural pathways when on trial drug versus placebo, and blood biomarkers, such as glucose, insulin, ghrelin, and cholecystokinin, are all potential markers. These markers individually or in combination could provide confirmatory support of efficacy. Similarly, in evaluating treatments for emotional outbursts the use of wearable devices of a sufficiently high quality to allow the calculation of heart rate variability (HRV) could be used in naturalistic settings as secondary outcome measures (see [52] for review). This would enable changes in HRV and other metrics to be measured. These, in turn, may provide an indication that the treatment concerned is likely to significantly reduce the risk of temper outbursts. FPWR, clinical research groups and the pharmaceutical industry could work in partnership to develop such approaches, and work with regulatory authorities to ensure that such measures are qualified and effectively incorporated into clinical trials.

Particularly with rare life-long conditions natural history studies are essential to understand the normal course of a disease and can play a key role providing a comparator against which those receiving a novel treatment can be judged. This can be particular important where the treatment in question may have a disease modifying effect impacting on more than one aspect of the phenotype at particular stages in development, and/or fundamentally impacting the developmental trajectory of the condition in question. Such natural history studies can inform trial design, enable the comparison of treatments, and allow for the study of syndrome sub-type variability in response. The use of data from the “Paving the way for Advances in Treatment and Health for PWS” study (PATH for PWS), a natural history study of PWS undertaken by FPWR, was an important component in the case supporting the approval of DCCR. HQ-CT scores at 26 and 52 weeks were significantly decreased in the treated group compared to a matched PATH for PWS comparison group, as were measures for other behaviours of concern. [74].

### Off-label use and repurposing of treatments

One of the most significant concerns expressed by families of children with PWS is the length of time trials can take and also both the costs of trials and the restrictions that come with funding through private equity. One option is to look to off-label prescribing and using existing medications repurposed for treating particular aspects of the PWS phenotype. This may be because the treatment approved for another condition may theoretical have an effect in PWS or incidentally the treatment has been observed to have an effect relevant to PWS. Also a specific drug used to treat a similar condition in the general population may be evaluate for use in people with PWS (see [106]). Existing examples include the evaluation of vagus nerve stimulation approved to treat epilepsy and depression to treat hyperphagia in PWS and then temper outbursts, pitolisant approved to treat narcolepsy in the general population to treat excessive day time sleepiness in PWS, guanfacine approved for treating ADHD for treating the emotional outbursts associated with PWS, or GLP-1 agonists approved to treat diabetes and/or obesity in the general population for treating hyperphagia in the PWS population.

### Sharing clinical trial experience and expertise

There is now a body of experience, mainly in the USA and to a lesser extent in Europe, which has developed as a result of previous and existing grant and industry-funded clinical trials. Mentoring, peer support and sharing of trial experience between participants with PWS, families, clinicians and those sponsoring the trial would be valuable. In the case of people with a rare neurodevelopmental condition, such as PWS, there are ethical and practical restrictions on the methodology used for clinical trials and special consideration needs to be given to issues of consent and the legalities specific to the countries where the trials are taking place. To avoid the possibility of the exploitation of those with neurodevelopmental conditions the intent of any trial must be for the benefit of people with that neurodevelopmental condition and that should be made clear. Third sector organisations, such as National PWS Associations, IPWSO and FPWR, all have an important role to play supporting the various parties undertaking research and clinical trials and ensuring that the rights of the participants are protected. IPWSO, given its global reach, has an important role in supporting the development of trial expertise in countries with limited trials experience. IPWSO and FPWR in partnership should host regular virtual research and trials meetings.

### Recruitment

The best strategies for recruitment for a clinical trial will depend upon the health service structure of each country, and diverse methods may be required. Where there is a well-established national rare diseases specialist reference centre, specialist interdisciplinary clinics, or a national register of people with PWS they may provide the main means of recruitment. For some countries care is provided at local level so information about people with PWS is not necessarily centralised. Under these circumstances the National PWS Association of that country may be the best means for promoting a trial and seeking participants. People with PWS will need at least one family or professional carer who is committed to the trial to actively support participation. Success in recruitment critically depends on establishing the most appropriate contacts in that country. This would include the National PWS Association, clinicians and those who provide support to people with PWS. Funding should be available to support families and professional carers and the trial design should minimise in-person attendance.

### Complexity of trials and the need for alternative approaches

Certain co-morbidities are common in people with PWS and have the potential to impact on any trial. In addition, changes in the family or social care environment may impact this risk and also the occurrence of the phenotypic characteristic that is the subject of the trial. For example, on reaching adulthood, leaving the structure of the home may lead to increased access to food and, as a result in weight increase and obesity. An increase in temper outbursts may be seen if there is a new member of support staff who is not familiar with how to minimise the risk of such outbursts. We recommend that such potential problems are considered when developing the methodology of the trial, and decisions be made as to whether particular co-morbidities and/or their treatments should be part of the exclusion criteria, or whether any complications that might arise can be addressed by other means, for example, statistically.

Three additional forms of data collection have the potential to be of value and useful secondary measures in trials of interventions in early childhood or of aspects of the neuropsychiatric phenotype in later life. These include: a) The use of N-of-1 studies in rare disorders to enable a more fine-grained approach to the investigation of clinically relevant outcomes or where a subset of participants have an unusual outcome [107], b) direct observation of eating behaviours and of other behaviours in naturalistic settings and also the use of carer completed diaries. Such approaches may provide a better understanding of the effectiveness of treatment in the person’s daily life and in their ordinary living environment; and c) qualitative interviews with family members, paid carers and the person with PWS so as to identify more subtle differences in response in those in the active treatment and in the placebo groups.

The requirement to demonstrate substantial evidence of efficacy applies to rare diseases and it is acknowledged that randomized double blind controlled studies are the gold standard. However, the amount of data that can be acquired is limited by the number of participants who can be recruited into trials, and this is even more challenging with the variability of participants and when the development programme targets a specific set of symptoms that are only relevant in one specific period in the life course of those with the syndrome. Considering the above challenges inherent in controlled trials in the targeted population, regulatory flexibility should be exercised in rare conditions, such as PWS, especially when the biological plausibility of the benefit is strong. In particular, flexibility to grant regulatory approvals based on biological plausibility despite the fact that the primary efficacy outcome does not reach statistical significance is a key topic to be debated. The challenge, of course, is to ensure that any treatment that is approved brings benefit that outweighs any associated risks. The use of registries, natural history or real-world data and data collected in specific programmes, such as compassionate use programmes, are important, further enabling the assessment of the benefit versus risk ratio. Overall, in rare and challenging neurodevelopmental conditions regulatory flexibility should be exercised, and the totality of evidence taken into account, to ultimately address high unmet medical needs.

We conclude on a positive note. We believe that the global third-sector (FPWR, IPWSO, National PWS Associations), people with PWS and their families, pharmaceutical companies and clinicians and researchers have developed strong and positive partnerships. There is a shared commitment to developing and seeing through to approval new treatments that have the potential to make a real difference in the lives of people with PWS. Such commitments are fostered through joint working, clinical and family conferences, and the establishment of strong research partnerships. One particular area of concern remains that is whether treatments, once approved, will then be funded by insurance companies or by state health services. Growth hormone, although approved in many countries over 20 years ago, is still not available universally. Here again National Associations, IPWSO and FPWR have an important advocacy role. Whilst each rare disorder is by definition rare, as a group rare disorders are common and on this basis, global organisations, such as IPWSO, Rare Diseases International, Eurordis, and Global Genes, are strongly supportive of the World Health Organisation’s campaign in support of the right of access to treatment regardless of a person’s ability to pay.

## Data Availability

Not applicable

## Footnotes

1 http://www.fpwr.org/pws-clinical-trial-consortium

2 https://www.citeline.com/pharmard?utm_source=google%26utm_medium=cpc%26utm_term=pharma%20r%26d%20annual%20review%202024|%26utm_campaign=ClinicalPharmaRD24EMEA%26gad_source=1%26gclid=EAIaIQobChMI8PjQ48vTiAMVtJRQBh3w4zpAEAAYASAAEgKHk_D_BwE.

## References

[1] European Organisation for Rare Diseases (EURORDIS). Rare diseases: understanding this public health priority. 2005. Available at: eurordis.org/IMG/pdf/princeps_document-EN.pdf. Accessed 20 Feb 2020.

[2] Protic D, Salcedo-Arellano MJ, Dy JB, Potter LA, Hagerman RJ. New targeted treatments for Fragile X syndrome. Curr Pediatr Rev. 2019;15(4):251–58. 10.2174/1573396315666190625110748. 31241016 10.2174/1573396315666190625110748 PMC6930353

[3] Furqan M. Trofinetide-a new chapter in Rett syndrome’s treatment. Front Pharmacol. 2023;16(14):1284035. 10.3389/fphar.2023.1284035. 10.3389/fphar.2023.1284035 PMC10687465 38035006

[4] Grugni G, Sartorio A, Crinò A. Growth hormone therapy for Prader-Willi syndrome: challenges and solutions. Ther Clin Risk Manag. 2016;2(12):873–81. 10.2147/TCRM.S70068. 10.2147/TCRM.S70068 PMC4898426 27330297

[5] Kimonis V, Surampalli A, Wencel M, Gold JA, Cowen NM. A randomized pilot efficacy and safety trial of diazoxide choline controlled-release in patients with Prader-Willi syndrome. PLoS One. 2019;14(9):e0221615. 10.1371/journal.pone.0221615. 31545799 10.1371/journal.pone.0221615 PMC6756513

[6] Miller JL, Bridges N, Felner EI, Salehi P, Yanovski JA, Stevenson DA, et al. Diazoxide choline extended-release tablets in Prader-Willi syndrome: a randomized, double-blind, withdrawal period study. J Clin Endocrinol Metab. 2026;2:dgaf661. 10.1210/clinem/dgaf661. 10.1210/clinem/dgaf661 PMC13183442 41482637

[7] Miller JL, Gevers E, Bridges N, Yanovski JA, Salehi P, Obrynba KS, et al. DESTINY PWS Investigators Diazoxide choline extended-release tablet in people with Prader-Willi syndrome: a Double-blind, placebo-controlled trial. J Clin Endocrinol Metab. 2023;108(7):1676–85. 10.1210/clinem/dgad014. 36639249 10.1210/clinem/dgad014 PMC10271219

[8] Miller JL, Gevers E, Bridges N, Yanovski JA, Salehi P, Obrynba KS, et al. C601/C602 Investigators. Diazoxide choline extended-release tablet in people with Prader-Willi syndrome: results from long-term open-label study. Obes (Silver Spring). 2024;32(2):252–61. 10.1002/oby.23928. 10.1002/oby.23928 PMC12181816 37919617

[9] Driscoll DJ, Miller JL, Schwartz S, et al. Prader-Willi syndrome. In: Adam MP, Mirzaa GM, Pagon RA, et al. In: GeneReviews® [Internet]. Seattle (WA): University of Washington, Seattle; 1998. 1993-2022. Available from: https://www.ncbi.nlm.nih.gov/books/NBK1330/. 20301505

[10] Bar C, Diene G, Molinas C, et al. Early diagnosis and care is achieved but should be improved in infants with Prader-Willi syndrome. Orphanet J Rare Dis. 2017;12:118. 10.1186/s13023-017-0673-6. 28659150 10.1186/s13023-017-0673-6 PMC5490212

[11] Whittington JE, Holland AJ, Webb T, Butler J, Clarke D, H B. Population prevalence and estimated birth incidence and mortality rate for people with Prader-Willi syndrome in one health district. J Med Genet. 2001;38:792–98. 11732491 10.1136/jmg.38.11.792 PMC1734766

[12] McCandless MS, Yin D, Yeh M, Czado S, Aghsaei S, Li JW, et al. SUN-604 U.S. Prevalence & mortality of Prader-Willi syndrome: a population-based study of medical claims. J Endocr Soc. 2020;4(Supplement_1):SUN-604. 10.1210/jendso/bvaa046.993.

[13] Pacoricona Alfaro DL, Lemoine P, Ehlinger V, Molinas C, Diene G, Valette M, et al. Causes of death in Prader-Willi syndrome: lessons from 11 years’ experience of a national reference center. Orphanet J Rare Dis. 2019;14(1):238. 10.1186/s13023-019-1214-2. 31684997 10.1186/s13023-019-1214-2 PMC6829836

[14] Whittington J, Holland A. Cognition in people with Prader-Willi syndrome: insights into genetic influences on cognitive and social development. Neurosci Biobehav Rev. 2017;72:153–67. 10.1016/j.neubiorev.2016.09.013. 27836461 10.1016/j.neubiorev.2016.09.013

[15] Schwartz L, Caixàs A, Dimitropoulos A, Dykens E, Duis J, Einfeld S, et al. Behavioral features in Prader-Willi syndrome (PWS): consensus paper from the International PWS Clinical Trial Consortium. J Neurodev Disord. 2021;13(1):25. 10.1186/s11689-021-09373-2. 10.1186/s11689-021-09373-2 PMC8215770 34148559

[16] Brown SSG, Manning KE, Fletcher P, Holland A. *In vivo* neuroimaging evidence of hypothalamic alteration in Prader-Willi syndrome. Brain Commun. 2022;4(5):fcac229. 10.1093/braincomms/fcac229. 36147452 10.1093/braincomms/fcac229 PMC9487704

[17] Tauber M, Hoybye C. Endocrine disorders in Prader-Willi syndrome: a model to understand and treat hypothalamic dysfunction. Lancet Diabetes Endocrinol. 2021;9(4):235–46. 10.1016/S2213-8587(21)00002-4. 33647242 10.1016/S2213-8587(21)00002-4

[18] Goldstone AP, Holland AJ, Hauffa BP, Hokken-Koelga AC, Tauber M. Recommendations for the diagnosis and management of Prader-Willi syndrome. J Clin Endocrinol Metab. 2008;93:4183–97. 18697869 10.1210/jc.2008-0649

[19] McCandless SE, Committee on Genetics. Clinical report-health supervision for children with Prader-Willi syndrome. Pediatrics. 2011;127(1):195–204. 10.1542/peds.2010-2820. Shawn E. 21187304 10.1542/peds.2010-2820

[20] Poitou C, Holland A, Höybye C, de Graaff LCG, Bottius S, Otterlei B, et al. The transition from pediatric to adult care in individuals with Prader-Willi syndrome. Endocr Connect. 2022;12(1):e220373. 10.1530/EC-22-0373. 36347048 10.1530/EC-22-0373 PMC9782397

[21] Dykens EM, Roof E, Hunt-Hawkins H. ‘The cure for us is a lot of things’: how young people with Prader-Willi syndrome view themselves and future clinical trials. J Appl Res Intellect Disabil. 2022;35(2):460–70. 10.1111/jar.12950. 34904341 10.1111/jar.12950

[22] Tsai JH, Scheimann AO, McCandless SE, Strong TV, Bridges JFP. Caregiver priorities for endpoints to evaluate treatments for Prader-Willi syndrome: a best-worst scaling. J Med Econ. 2018;21(12):1230–37. 10.1080/13696998.2018.1528980. 30256699 10.1080/13696998.2018.1528980

[23] Bellis SA, Kuhn I, Adams S, Mullarkey L, Holland A. The consequences of hyperphagia in people with Prader-Willi syndrome: a systematic review of studies of morbidity and mortality. Eur J Med Genet. 2022;65(1):104379. 10.1016/j.ejmg.2021.104379. 34748997 10.1016/j.ejmg.2021.104379

[24] Rosenberg AGW, Wellink CM, Tellez Garcia JM, Pellikaan K, Van Abswoude DH, Davidse K, et al. Health problems in adults with Prader-Willi syndrome of different genetic subtypes: cohort study, meta-analysis and review of the literature. J Clin Med. 2022;11(14):4033. 10.3390/jcm11144033. 35887798 10.3390/jcm11144033 PMC9323859

[25] Dempsey D, Hall M, Lanning B, Barron-Millar B, Huang M, Cowen N, et al. The burden of illness in Prader-Willi syndrome: a systematic literature review. Orphanet J Rare Dis. 2025;20(1):374. 10.1186/s13023-025-03787-0. 40708003 10.1186/s13023-025-03787-0 PMC12291511

[26] Micallef Pulè K, Hughes BM. Anxiety, depression and stress in parents and siblings of people who have Prader-Willi syndrome: morbidity prevalence and mitigating factors. J Intellect Disabil Res. 2025;69(5):417–27. 10.1111/jir.13223. 39970479 10.1111/jir.13223 PMC11966354

[27] Kayadjanian N, Schwartz L, Farrar E, Comtois KA, Strong TV. High levels of caregiver burden in Prader-Willi syndrome. PLoS One. 2018;13(3):e0194655. 10.1371/journal.pone.0194655. 29579119 10.1371/journal.pone.0194655 PMC5868812

[28] Kayadjanian N, Vrana-Diaz C, Bohonowych J, Strong TV, Morin J, Potvin D, et al. Characteristics and relationship between hyperphagia, anxiety, behavioral challenges and caregiver burden in Prader-Willi syndrome. PLoS One. 2021;16(3):e0248739. 10.1371/journal.pone.02487392. 33765021 10.1371/journal.pone.0248739 PMC7993772

[29] López-Bastida J, Linertová R, Oliva-Moreno J, Posada-de-la-Paz M, Serrano-Aguilar P, Kanavos P, et al. Social/Economic costs and health-related quality of life in patients with Prader-Willi syndrome in Europe.Eur. J Health Econ. 2016;17 Suppl 1:99–108. 10.1007/s10198-016-0788-z. 10.1007/s10198-016-0788-z 27038627

[30] Wetmore DZ, Garner CC. Emerging pharmacotherapies for neurodevelopmental disorders. J Dev Behav Pediatr. 2010;31(7):564–81. 10.1097/DBP.0b013e3181ee3833. 20814256 10.1097/DBP.0b013e3181ee3833 PMC2967570

[31] Sahoo T, Del Gaudio D, German JR, Shinawi M, Peters SU, Person RE, et al. Prader-Willi phenotype caused by paternal deficiency for the HBII-85 C/D box small nucleolar RNA cluster. Nat Genet. 2008;40(6):719–21. 10.1038/ng.158. 18500341 10.1038/ng.158 PMC2705197

[32] Duker AL, Ballif BC, Bawle EV, Person RE, Mahadevan S, Alliman S, et al. Paternally inherited microdeletion at 15q11.2 confirms a significant role for the SNORD116 C/D box snoRNA cluster in Prader-Willi syndrome. Eur J Hum Genet. 2010;18(11):1196–201. 10.1038/ejhg.2010.102. 20588305 10.1038/ejhg.2010.102 PMC2987474

[33] Kim SJ, Miller JL, Kuipers PJ, German JR, Beaudet AL, Sahoo T, et al. Unique and atypical deletions in Prader-Willi syndrome reveal distinct phenotypes. Eur J Hum Genet. 2012;20(3):283–90. 10.1038/ejhg.2011.187. 22045295 10.1038/ejhg.2011.187 PMC3283188

[34] Zahova S, Isles AR. Animal models for Prader-Willi syndrome. Handb Clin Neurol. 2021;181:391–404. 10.1016/B978-0-12-820683-6.00029-4. 34238473 10.1016/B978-0-12-820683-6.00029-4

[35] Althammer F, Muscatelli F, Grinevich V, Schaaf CP. Oxytocin-based therapies for treatment of Prader-Willi and Schaaf-Yang syndromes: evidence, disappointments, and future research strategies. Transl Psychiatry. 2022;12(1):318. 10.1038/s41398-022-02054-1. 35941105 10.1038/s41398-022-02054-1 PMC9360032

[36] Huang WK, Wong SZH, Pather SR, Nguyen PTT, Zhang F, Zhang DY, et al. Generation of hypothalamic arcuate organoids from human induced pluripotent stem cells. Cell STEM Cell. 2021;28(9):1657–70.e10. 10.1016/j.stem.2021.04.006. 33961804 10.1016/j.stem.2021.04.006 PMC8419002

[37] Fandakova Y, Hartley CA. Mechanisms of learning and plasticity in childhood and adolescence. Dev Cogn Neurosci. 2020;42:100764. 10.1016/j.dcn.2020.100764. 32072937 10.1016/j.dcn.2020.100764 PMC7013153

[38] Hessl D, Sansone SM, Berry-Kravis E, Riley K, Widaman KF, Abbeduto L, et al. The NIH Toolbox Cognitive Battery for intellectual disabilities: three preliminary studies and future directions. J Neurodev Disord. 2016;8(1):35. 10.1186/s11689-016-9167-4 27602170 10.1186/s11689-016-9167-4 PMC5012003

[39] Mullen EM. Mullen scales of early learning (AGS ed.). American Guidance Service, Inc; 1995.

[40] Sparrow SS, Cicchetti DV, Saulnier CA. Vineland Adaptive behavior scales. 3rd ed. Bloomington, MN: Pearson Assessment; 2016.

[41] Wechsler D. Manual for the Wechsler intelligence scale for children-revised. Psychological Corporation; 1974.

[42] Wechsler D. WAIS-R: Wechsler adult intelligence scale-revised. New York, N.Y: Psychological Corporation; 1981.

[43] Rutter M, Bailey A, Lord C. The social communication questionnaire. Western Psychological Services; 2003.

[44] Constantino JN, Davis SA, Todd RD, Schindler MK, Gross MM, Brophy SL, et al. Social responsiveness scale [Database record]. APA PsycTests; 2003. 10.1037/t17260-000.

[45] Aman MG, Singh NN, Stewart AW, Field CJ. Psychometric characteristics of the Aberrant Behavior Checklist. Am J Ment Deficiency. 1985;89(5):492–502. 3158201

[46] Yudofsky SC, Silver JM, Jackson W, Endicott J, Williams D. The Overt Aggression Scale for the objective rating of verbal and physical aggression. Am J Psychiatry. 1986;143(1):35–39. 3942284 10.1176/ajp.143.1.35

[47] Cotter SP, Schwartz L, Strong TV, Bender RH, Fehnel SE. The Prader-Willi syndrome anxiousness and distress behaviors questionnaire: development and psychometric validation. Value Health. 2023;26(2):243–50. 36202701 10.1016/j.jval.2022.08.004

[48] Bodfish JW, Symons FJ, Parker DE, Lewis MH. Repetitive behavior scale-revised (RBS-R) [Database record]. APA PsycTests; 2000. 10.1037/t17338-000.

[49] Dykens EM, Roof E, Hunt-Hawkins H. The Prader-Willi syndrome Profile: validation of a new measure of behavioral and emotional problems in Prader-Willi syndrome. Orphanet J Rare Dis. 2024;19(1):83. 10.1186/s13023-024-03045-9. 38395848 10.1186/s13023-024-03045-9 PMC10885615

[50] Iwata BA, Pace GM, Kissel RC, Nau PA, Farber JM. The Self-Injury Trauma (SIT) Scale: a method for quantifying surface tissue damage caused by self-injurious behavior. J Appl Behav Anal. 1990;23(1):99–110. 10.1901/jaba.1990.23-99. 2335488 10.1901/jaba.1990.23-99 PMC1286214

[51] Johns MW. A new method for measuring daytime sleepiness: the Epworth sleepiness scale. Sleep J Sleep Res Sleep Med. 1991;14(6):540–45. 10.1093/sleep/14.6.540. 10.1093/sleep/14.6.540 1798888

[52] Izmailova ES, Wagner JA, Perakslis ED. Wearable devices in clinical trials: hype and hypothesis. Clin Pharmacol Ther. 2018;104(1):42–52. 10.1002/cpt.966. 29205294 10.1002/cpt.966 PMC6032822

[53] Forrest CB, Devine J, Bevans KB, et al. Development and psychometric evaluation of the PROMIS pediatric life satisfaction item banks, child-report, and parent-proxy editions. Qual Life Res. 2018;27(1):217–34. 28828568 10.1007/s11136-017-1681-7 PMC5771844

[54] Dykens EM, Roof E, Hunt-Hawkins H. Cognitive and adaptive advantages of growth hormone treatment in children with Prader-Willi syndrome. J Child Psychol Psychiatry. 2017;58(1):64–74. 10.1111/jcpp.12601. 27481444 10.1111/jcpp.12601 PMC5161611

[55] Trueba-Timmermans DJ, Grootjen LN, Juriaans AF, Mahabier EF, Kerkhof GF, Rings EHHM, et al. Cognitive function during 3 years of growth hormone in previously growth hormone-treated young adults with Prader-Willi syndrome. Eur J Endocrinol. 2023;189(1):132–39. 10.1093/ejendo/lvad084. 37440711 10.1093/ejendo/lvad084

[56] Kim Y, Wang SE, Jiang YH. Epigenetic therapy of Prader-Willi syndrome. Transl Res. 2019;208:105–18. 10.1016/j.trsl.2019.02. 30904443 10.1016/j.trsl.2019.02.012 PMC6527448

[57] Kwon EJ, Kim YJ. What is fetal programming?: a lifetime health is under the control of in utero health. Obstet Gynecol Sci. 2017, Nov;60(6):506–19. 10.5468/ogs.2017.60.6.506. 29184858 10.5468/ogs.2017.60.6.506 PMC5694724

[58] Tauber M, Boulanouar K, Diene G, Çabal-Berthoumieu S, Ehlinger V, Fichaux-Bourin P, et al. The use of oxytocin to improve feeding and social skills in infants with Prader-Willi syndrome. Pediatrics. 2017;139(2):e20162976. 10.1542/peds.2016-2976. 28100688 10.1542/peds.2016-2976

[59] Swaab DF, Purba JS, Hofman MA. Alterations in the hypothalamic paraventricular nucleus and its oxytocin neurons (putative satiety cells) in Prader-Willi syndrome: a study of five cases. J Clin Endocrinol Metab. 1995;80(2):573–79. 10.1210/jcem.80.2.7852523. 7852523 10.1210/jcem.80.2.7852523

[60] Meziane H, Schaller F, Bauer S, Villard C, Matarazzo V, Riet F, et al. An early postnatal oxytocin treatment prevents social and learning deficits in adult mice deficient for Magel2, a gene involved in Prader-Willi syndrome and autism. Biol Psychiatry. 2015;78(2):85–94. 10.1016/j.biopsych.2014.11.010. 25599930 10.1016/j.biopsych.2014.11.010

[61] Schaller F, Watrin F, Sturny R, Massacrier A, Szepetowski P, Muscatelli F. A single postnatal injection of oxytocin rescues the lethal feeding behaviour in mouse newborns deficient for the imprinted Magel2 gene. Hum Mol Genet. 2010,;19(24):4895–905. 10.1093/hmg/ddq424. 20876615 10.1093/hmg/ddq424

[62] Gigliucci V, Busnelli M, Santini F, Paolini C, Bertoni A, Schaller F, et al. Oxytocin receptors in the Magel2 mouse model of autism: specific region, age, sex and oxytocin treatment effects. Front Neurosci. 2023;14(17):1026939. 10.3389/fnins.2023.1026939. 10.3389/fnins.2023.1026939 PMC10043208 36998737

[63] Mahmoud R, Kimonis V, Butler MG. Clinical trials in Prader-Willi syndrome: a review. Int J Mol Sci. 2023, Jan, 21;24(3):2150. 10.3390/ijms24032150. 36768472 10.3390/ijms24032150 PMC9916985

[64] Beaulieu K, Blundell J. The psychobiology of hunger - a scientific perspective. Topoi. 2021;40:565–74. 10.1007/s11245-020-097724-z.

[65] Holsen LM, Savage CR, Martin LE, Bruce AS, Lepping RJ, Ko E, et al. Importance of reward and prefrontal circuitry in hunger and satiety: Prader-Willi syndrome vs simple obesity. Int J Obes (Lond). 2012;36(5):638–47. 10.1038/ijo.2011.204. 22024642 10.1038/ijo.2011.204 PMC3270121

[66] Hinton EC, Holland AJ, Gellatly MS, Soni S, Patterson M, Ghatei MA, et al. Neural representations of hunger and satiety in Prader-Willi syndrome. Int J Obes (Lond). 2006;30(2):313–21. 10.1038/sj.ijo.0803128. 16276365 10.1038/sj.ijo.0803128

[67] Zhang Y, Wang J, Zhang G, Zhu Q, Cai W, Tian J, et al. The neurobiological drive for overeating implicated in Prader-Willi syndrome. Brain Res. 2015;1620:72–80. 10.1016/j.brainres.2015.05.008. 25998539 10.1016/j.brainres.2015.05.008

[68] Cummings DE, Clement K, Purnell JQ, Vaisse C, Foster KE, Frayo RS, et al. Elevated plasma ghrelin levels in Prader Willi syndrome. Nat Med. 2002;8(7):643–44. 10.1038/nm0702-643. 12091883 10.1038/nm0702-643

[69] DelParigi A, Tschöp M, Heiman ML, Salbe AD, Vozarova B, Sell SM, et al. High circulating ghrelin: a potential cause for hyperphagia and obesity in Prader-Willi syndrome. J Clin Endocrinol Metab. 2002;87(12):5461–64. 10.1210/jc.2002-020871. 12466337 10.1210/jc.2002-020871

[70] Millendo Therapeutics SAS. A Phase 2b/3 study to evaluate the safety, tolerability, and effects of livoletide (AZP-531), an unacylated ghrelin analogue, on food-related behaviors in patients with Prader-Willi syndrome. Available online: https://clinicaltrials.gov/ct2/show/NCT03790865. Accessed 1 Nov 2022.

[71] Allas S, Caixàs A, Poitou C, Coupaye M, Thuilleaux D, Lorenzini F, et al. AZP-531, an unacylated ghrelin analog, improves food-related behavior in patients with Prader-Willi syndrome: a randomized placebo-controlled trial. PLoS One. 2018;13(1):e0190849. 10.1371/journal.pone.0190849. 29320575 10.1371/journal.pone.0190849 PMC5761957

[72] McCandless SE, Yanovski JA, Miller J, Fu C, Bird LM, Salehi P, et al. Effects of MetAP2 inhibition on hyperphagia and body weight in Prader-Willi syndrome: a randomized, double-blind, placebo-controlled trial. Diabetes Obes Metab. 2017;19(12):1751–61. 10.1111/dom.13021. 28556449 10.1111/dom.13021 PMC5673540

[73] Roof E, Deal CL, McCandless SE, Cowan RL, Miller JL, Hamilton JK, et al. Intranasal carbetocin reduces hyperphagia, anxiousness, and distress in Prader-Willi syndrome: CARE-PWS Phase 3 trial. J Clin Endocrinol Metab. 2023;108(7):1696–708. 10.1210/clinem/dgad015. 36633570 10.1210/clinem/dgad015 PMC10271225

[74] Strong TV, Miller JL, McCandless SE, Gevers E, Yanovski JA, Matesevac L, et al. Behavioral changes in patients with Prader-Willi syndrome receiving diazoxide choline extended-release tablets compared to the PATH for PWS natural history study. J Neurodev Disord. 2024;16(1):22. 10.1186/s11689-024-09536-x. 38671361 10.1186/s11689-024-09536-x PMC11046911

[75] Tsai JH, Crossnohere NL, Strong T, Bridges JFP. Measuring meaningful benefit-risk tradeoffs to promote patient-focused drug development in Prader-Willi syndrome: a discrete-choice experiment. MDM Policy Pract. 2021;6(2):23814683211039457. 10.1177/23814683211039457. 34497876 10.1177/23814683211039457 PMC8419554

[76] Tunnicliffe P, Woodcock K, Bull L, Oliver C, Penhallow J. Temper outbursts in Prader-Willi syndrome: causes, behavioural and emotional sequence and responses by carers. J Intellect Disabil Res. 2014;58(2):134–50. 10.1111/jir.12010. 23374136 10.1111/jir.12010

[77] Deest M, Wieting J, Jakob MM, Deest-Gaubatz S, Groh A, Seifert J, et al. Aripiprazole treatment for temper outbursts in Prader-Willi syndrome. Orphanet J Rare Dis. 2022;17(1):324. 10.1186/s13023-022-02470-y. 36028863 10.1186/s13023-022-02470-y PMC9419314

[78] Deest M, Jakob MM, Seifert J, Bleich S, Frieling H, Eberlein C. Sertraline as a treatment option for temper outbursts in Prader-Willi syndrome. Am J Med Genet A. 2021;185(3):790–97. 10.1002/ajmg.a.62041. 33369086 10.1002/ajmg.a.62041

[79] Singh D, Silver M, Jacob T. A randomized double-blind placebo-controlled trial of guanfacine extended release for aggression and self-injurious behavior associated with Prader-Willi syndrome. Am J Med Genet B Neuropsychiatr Genet. 2025;198(6):62–70. 10.1002/ajmg.b.33032. 40395104 10.1002/ajmg.b.33032

[80] Manning KE, McAllister CJ, Ring HA, Finer N, Kelly CL, Sylvester KP, et al. Novel insights into maladaptive behaviours in Prader-Willi syndrome: serendipitous findings from an open trial of vagus nerve stimulation. J Intellect Disabil Res. 2016;60(2):149–55. 10.1111/jir.12203. 26018613 10.1111/jir.12203 PMC4950305

[81] Manning KE, Beresford-Webb JA, Aman LCS, Ring HA, Watson PC, Porges SW, et al. Transcutaneous vagus nerve stimulation (t-VNS): a novel effective treatment for temper outbursts in adults with Prader-Willi syndrome indicated by results from a non-blind study. PLoS One. 2019;14(12):e0223750. 10.1371/journal.pone.0223750. 31794560 10.1371/journal.pone.0223750 PMC6890246

[82] Schmausser M, Holland A, Beresford-Webb J, Eglen SJ, Manning K, Aman L, et al. Effects of long-term transcutaneous auricular vagus nerve stimulation on circadian vagal activity in people with Prader-Willi syndrome: a case-series. Res Dev Disabil. 2024;13(154):104855. 10.1016/j.ridd.2024.104855. 10.1016/j.ridd.2024.104855 39405838

[83] Butler MG, Victor AK, Reiter LT. Autonomic nervous system dysfunction in Prader-Willi syndrome. Clin Auton Res. 2023;33(3):281–86. 10.1007/s10286-022-00909-7. 36515769 10.1007/s10286-022-00909-7

[84] Holland A, Manning K. t-VNS to treat disorders of behaviour in Prader-Willi syndrome and in people with other neurodevelopmental conditions. Auton Neurosci. 2022;239:102955. 10.1016/j.autneu.2022.102955. 35219158 10.1016/j.autneu.2022.102955

[85] Hall SS, Hustyi KM, Chui C, Hammond JL. Experimental functional analysis of severe skin-picking behavior in Prader-Willi syndrome. Res Dev Disabil. 2014;35(10):2284–92. 10.1016/j.ridd.2014.05.025. 24952370 10.1016/j.ridd.2014.05.025

[86] Shapira NA, Lessig MC, Murphy TK, Driscoll DJ, Goodman WK. Topiramate attenuates self-injurious behaviour in Prader-Willi syndrome. Int J Neuropsychopharmacol. 2002;5(2):141–45. 10.1017/S1461145702002833. 12135538 10.1017/S1461145702002833

[87] Wieting J, Deest M, Bleich S, Frieling H, Eberlein C. N-Acetylcysteine provides limited efficacy as treatment option for skin picking in Prader-Willi syndrome. Am J Med Genet A. 2022;188(3):828–35. 10.1002/ajmg.a.62589 34854203 10.1002/ajmg.a.62589

[88] Smathers SA, Wilson JG, Nigro MA. Topiramate effectiveness in Prader-Willi syndrome. Pediatr Neurol. 2003;28(2):130–33. 10.1016/s0887-8994(02)00490-3. 12699864 10.1016/s0887-8994(02)00490-3

[89] Duis J, Pullen LC, Picone M, Friedman N, Hawkins S, Sannar E, et al. Diagnosis and management of sleep disorders in Prader-Willi syndrome. J Clin Sleep Med. 2022;18(6):1687–96. 10.5664/jcsm.9938. 35172921 10.5664/jcsm.9938 PMC9163612

[90] Revana A, Bhattacharjee R, Miller JL, et al. A proof-of-concept study of pitolisant for excessive daytime sleepiness in patients with Prader-Willi syndrome. J Clin Sleep Med. 2025;21(11):1893–902. 40605372 10.5664/jcsm.11800 PMC12582198

[91] Dimitropoulos A, Zyga O, Doernberg E, Russ SW. Show me what happens next: preliminary efficacy of a remote play-based intervention for children with Prader-Willi syndrome. Res Dev Disabil. 2021;108:103820. 10.1016/j.ridd.2020.103820. 33307337 10.1016/j.ridd.2020.103820

[92] Hughes BM, Holland A, Hödebeck-Stuntebeck N, Garrick L, Goldstone AP, Lister M, et al. Body weight, behaviours of concern, and social contact in adults and adolescents with Prader-Willi syndrome in full-time care services: findings from pooled international archival data. Orphanet J Rare Dis. 2024;19(1):48. 10.1186/s13023-024-03035-x. 38326873 10.1186/s13023-024-03035-x PMC10848374

[93] Gordon BG. Vulnerability in research: basic ethical concepts and general approach to review. Ochsner J. 2020;20(1):34–38. 10.31486/toj.19.0079. 32284680 10.31486/toj.19.0079 PMC7122263

[94] Dykens EM, Roof E, Hunt-Hawkins H, Strong TV. Validation of the food safe zone questionnaire for families of individuals with Prader-Willi syndrome. J Neurodev Disord. 2024;17:6. 10.1186/s11689-024-09589-y PMC11806870 39923017

[95] Fehnel S, Brown M, Nelson L, Chen A, Roof E, Kim DD, et al. Development of the hyperphagia questionnaire for use in Prader-Willi syndrome clinical trials. Value Health. 2015;18:A25–25. 10.1016/j.jval.2015.03.154.

[96] Schwartz L, Vrana-Diaz CJ, Bohonowych JE, Matesevac L, Strong TV. Life satisfaction, global health and mood in Prader-Willi syndrome: use of PROMIS and the Glasgow depression scale. J Appl Res Intell Disabil. 2025;38(2):e70053. 10.1111/jar.70053 PMC11995245 40223576

[97] Fujiura GT. RRTC Expert Panel on Health Measurement Self-reported health of people with intellectual disability. Intellect Dev Disabil. 2012;50(4):352–69. 10.1352/1934-9556-50.4.352. 22861136 10.1352/1934-9556-50.4.352

[98] Scott HM, Havercamp SM. Comparisons of self and proxy report on health-related factors in people with intellectual disability. J Appl Res Intellect Disabil. 2018;31(5):927–36. 10.1111/jar.12452. 29608230 10.1111/jar.12452

[99] Dykens EM, Maxwell MA, Pantino E, Kossler R, Roof E. Assessment of hyperphagia in Prader-Willi syndrome. Obes (Silver Spring). 2007;15(7):1816–26. 10.1038/oby.2007.216. 10.1038/oby.2007.216 17636101

[100] Matesevac L, Vrana-Diaz CJ, Bohonowych JE, Schwartz L, Strong TV. Analysis of Hyperphagia Questionnaire for Clinical Trials (HQ-CT) scores in typically developing individuals and those with Prader-Willi syndrome. Sci Rep. 2023;13(1):20573. 10.1038/s41598-023-48024-5. 37996659 10.1038/s41598-023-48024-5 PMC10667498

[101] Leffler M, Elder SJ, Bolding S, Hefner M, Miller JL, et al. A qualitative methodology to support the evaluation of novel treatments for hyperphagia in people with Prader-Willi syndrome. Int J Rare Dis Disord. 2021;4:030. 10.23937/2643-4571/1710030.

[102] Anikwe CV, Nweke HF, Ikegwu AC, Egwuonwu CA, Onu FU, Alo UR, et al. Mobile and wearable sensors for data-driven health monitoring system: state-of-the-art and future prospect. Expert Syst Appl. 2022;202:117362. 10.1016/j.eswa.2022.117362.

[103] Martin L, Hutchens M, Hawkins C, et al. How much do clinical trials cost? Nat Rev Drug Discov. 2017;16:381–82. 10.1038/nrd.2017.70. 28529317 10.1038/nrd.2017.70

[104] Nisar S, Haris M. Neuroimaging genetics approaches to identify new biomarkers for the early diagnosis of autism spectrum disorder. Mol Psychiatry. 2023;28(12):4995–5008. 10.1038/s41380-023-02060-9. Erratum in: Mol Psychiatry. 2023;28(12):5009–5010. doi 10.1038/s41380-023-02115-x. 10.1038/s41380-023-02060-9 PMC11041805 37069342

[105] Robinson E, Bevelander KE, Field M, Jones A. Methodological and reporting quality in laboratory studies of human eating behavior. Appetite. 2018;1(125):486–91. 10.1016/j.appet.2018.02.008. 10.1016/j.appet.2018.02.008 PMC5890731 29452224

[106] Chakhtoura M, Haber R, Ghezzawi M, Rhayem C, Tcheroyan R, Mantzoros CS. Pharmacotherapy of obesity: an update on the available medications and drugs under investigation. EClinicalMedicine. 2023;58:101882. 10.1016/j.eclinm.2023.101882. 36992862 10.1016/j.eclinm.2023.101882 PMC10041469

[107] Müller AR, Brands MMMG, van de Ven PM, Roes KCB, Cornel MC, van Karnebeek CDM, et al. Systematic review of N-of-1 studies in rare genetic neurodevelopmental disorders: the power of 1. Neurology. 2021;96(11):529–40. 10.1212/WNL.0000000000011597. 33504638 10.1212/WNL.0000000000011597 PMC8032375

